# Fertility Inhibitor Heterobimetallic Complexes of Platinum(II) and Palladium(II): Synthetic, Spectroscopic and Antimicrobial Aspects

**DOI:** 10.1155/MBD.2000.105

**Published:** 2000

**Authors:** Kripa Sharma, S. C. Joshi, R. V. Singh

**Affiliations:** 1 Department of Chemistry University of Rajasthan Jaipur 302004 India; 2 Department of Zoology University of Rajasthan Jaipur 302004 India

## Abstract

Synthetic, spectroscopic and antimicrobial aspects of some fertility inhibitor heterobimetallic complexes have been carried out. These heterobimetallic chelates [M(C_5_H_5_N_3_)_2_M_2_'(R)_4_]Cl_2_ (M = Pd or Pt and M' = Si, Sn, Ti and Zr) have been successfully synthesinzed via the reaction of M(C_5_H_7_N_3_)_2_Cl_2_ with group four or fourteen dichlorides in 1:2 stoichiometric proportions. The products were characterized by elemental analyses, molecular weight determinations, magnetic susceptibility measurements, conductance, and IR multinuclear NMR and electronic spectral studies. A square planar geometry has been suggested for all the complexes with the help of spectral data. Conductivity data strongly suggest that chlorine atoms are ionic in nature due to which complexes behave as electrolytes. All the complexes have been evaluated for their antmicrobial effects on different species of pathogenic fungi and bacteria. The testicular sperm density, testicular sperm morphology, sperm motility, density of cauda epididymal spermatozoa and fertility in mating trails and biochemical parameters of reproductive organs have been examined and discussed.


### FERTILITY INHIBITOR HETEROBIMETALLIC COMPLEXES OF PLATINUM(II) AND PALLADIUM(II): SYNTHETIC, SPECTROSCOPIC AND ANTIMICROBIAL ASPECTS

Kripa Sharma[1], S. C. Joshi[2] and R.V. Singh*[1]

University of Rajasthan, Jaipur 302004, India [1] Department of Chemistry [2] Department of Zoology

### ABSTRACT

Synthetic, spectroscopic and antimicrobial aspects of some fertility inhibitor heterobimetallic complexes have been carried out. These heterobimetallic chelates $[M(C_5H_5N_3)_2M_2'(R)_4]Cl_2$ (M = Pd or Pt and M' = Si, Sn, Ti and Zr) have been successfully synthesized via the reaction of $M(C_5H_7N_3)_2Cl_2$ with group four or fourteen dichlorides in 1:2 stoichiometric proportions. The products were characterized by elemental analyses, molecular weight determinations, magnetic susceptibility measurements, conductance, and IR, multinuclear NMR and electronic spectral studies. A square planar geometry has been suggested for all the complexes with the help of spectral data. Conductivity data strongly suggest that chlorine atoms are ionic in nature due to which complexes behave as electrolytes. All the complexes have been evaluated for their antmicrobial effects on different species of pathogenic fungi and bacteria. The testicular sperm density, testicular sperm morphology, sperm motility, density of cauda epididymal spermatozoa and fertility in mating trails and biochemical parameters of reproductive organs have been examined and discussed.

### INTRODUCTION

The complexes of metal ions with macrocyclic ligands are significant because of their resemblance with many natural systems, such as porphyrins[1] and cobalamines[2]. Many of the transition metal ions in the living systems work as enzymes or carriers in macrocyclic ligand field environment. Therefore, meaningful research in this direction might generate simple models for biologically occurring metallo enzymes[3] and thus will help in developing our understanding of biological systems. These ligands are also of theoretical interest as they are capable of furnishing an environment of controlled geometry and ligand field strength[4-7]. A literature survey disclosed that a number of polydentate macrocyclic ligands and their metal complexes have been reported[8]. Their electronic properties and reactivities are ressemble those of porphyrins.

On the other hand, the discovery of cisplatin, an important cytotoxic drug used in the treatment of a variety of human cancer, has given a new dimention to the coordination chemistry of platinum and palladium. It may be possible that useful anticancer drugs might be found among palladium complexes. A wealth of data has been accumulated in the search of novel potent antitumor agents. Transition metal chelates of nitrogen donor ligands have been extensively screened for their antitumor activity. Most of them showed some activity but significant activities were exhibited by palladium and platinum complexes. In principle, coordination compounds offer a great variety of shapes and reactivities for use in drug design, the most detailed advances have been in understanding how cis-DDP binds to DNA. A clear knowledge of how platinum antitumor drugs work would have major implications for the further design and improvement of these inorganic drugs. The use of metal as template in condensation reactions has led to the synthesis of a large number of metal complexes of macrocyclic ligands. Macrocyclic ligands are relatively rigid and thus impose a specific coordination geometry on the metal ion[9]. Macrocyclic ligands display a number of chemical interesting features which include unusual structures. The occurrence of metal exchange (transmetallation) reactions is used for the preparation of new metal complexes not accessible by direct synthetic procedure[10].

A wider range of organosilicon compounds[11] is being studied due to their importance in resin and liquid polymer Chemistry[12,13] Well known exemplary series of di and triorganotin halides with various nitrogen and oxygen/sulphur containing ligands[14,15] have been found to possess significant biological and pharmacological activities. Screening data for tin derivatives [16,17] have revealed that many more diorganotin compounds exhibit antitumour activity than the corresponding mono-,tri-,and tetra-organotins or the inorganic tin chemicals, while within the diorganotin class, the highest activity was given by the diethyl tin derivatives[18]. Similarly, some of the organotin have been evaluated as potential mosquito larvicides[19]. Transition metal complexes of nitrogen donor ligands have been studied in detail on account of their interesting stereochemistry and wide practical utility.[20,21] Transition metals and their complexes have evolved great interest due to their biological potential[22,23] unusual structural aspects, unique stereo and magneto chemistry [24] and ability to form multiple complexes.

Several reports have appeared on multimetallic complexes[25] associated with electrochemical, magnetic and spectroscopic studies and their stable complexes which are of biological interest but studies on the heterobimetallic complexes of palladium and platinum seem to be comparatively limited. Therefore, in view of the above facts it was considered as useful to synthesize such a type of compounds with an aim to characterize them structurally, electrochemically and biologically.

### MATERIALS AND METHODS

All the chemicals and solvents used were dried and purified by standard methods. The reactions were carried out under strictly anhydrous conditions.

**Preparation of $M(C_5H_7N_3)Cl_2$**

The solution of $MCl_2$ (1 mmol) was mixed with the methanolic solution of 2,6 diaminopyridine (2 mmol). The reaction mixture was stirred for 6-7 hours. The resulting precipitate was recovered by filteration washed with methanol and dried in vacuo. The reactions proceed as: $MCl_2 + 2C_5H_7N_3 \rightarrow M(C_5H_7N_3)_2Cl_2$.

**Preparation of Heterobimetallic Complexes**

Heterobimetallic complexes were synthesized by stirring a methanolic solution of $M(C_5H_7N_3)_2Cl_2$ with methanolic solution of group four or fourteen metal dichlorides $Cp_2M'Cl_2$, $Ph_2MCl_2$ and $Me_2MCl_2$. The reaction mixture was kept at room temperature for overnight and stirred for 6-7 hours to complete the reaction. The coloured solid compound separated out. The excess of the solvent was removed under reduced pressure and the complexes were dried for 3-4 hours in vacuo. The complexes were purified by crystallization. The yield was 60-70%. The analytical and physical data of the isolated precursor and their complexes are given in Table I.

**Table I: Physical Properties and Analytical Data of Complexes**

| Complex | Colour | M.P. (°C) | Analysis(%) M Found (Calcd) | Pt/Pd Found (Calcd) | N Found (Calcd) | Cl Found (Calcd) | Mol.Wt. Found (Calcd) |
|---|---|---|---|---|---|---|---|
| $Pd(C_5H_7N_3)_2Cl_2$ | Light Brown | 110 (d) | - | 26.90 (26.88) | 21.24 (21.21) | 17.92 (17.90) | 395.56 (371.69) |
| $Pd(C_5H_5N_3)_2Cl_2Sn_2(CH_3)_4$ | Orange | 101 | 15.44 (15.42) | 34.45 (34.43) | 12.19 (12.14) | 10.28 (10.25) | 689.02 (680.00) |
| $Pd(C_5H_5N_3)_2Cl_2Sn_2(C_6H_5)_4$ | Reddish Brown | 126 | 11.35 (11.32) | 25.32 (25.30) | 8.96 (8.93) | 7.56 (7.54) | 937.32 (922.34) |
| $Pd(C_5H_5N_3)_2Cl_2Si_2(CH_3)_4$ | Dark Brown | 174 | 20.94 (20.93) | 11.05 (11.03) | 16.54 (16.52) | 13.96 (13.93) | 507.80 (489.84) |
| $Pd(C_5H_5N_3)_2Cl_2Si_2(C_6H_5)_4$ | Red | 152 (d) | 14.07 (14.04) | 14.85 (14.82) | 11.11 (11.09) | 9.37 (9.35) | 756.06 (734.87) |
| $Pd(C_5H_5N_3)_2Cl_2Ti_2(C_5H_5)_4$ | Light Brown | >300 | 14.23 (14.00) | 12.80 (12.78) | 11.24 (11.21) | 9.48 (9.45) | 747.66 (723.78) |
| $Pd(C_5H_5N_3)_2Cl_2Zr_2(C_5H_5)_4$ | Dark Green | 120 | 12.75 (12.73) | 21.86 (21.83) | 10.07 (10.04) | 8.49 (8.47) | 834.34 (829.38) |
| $Pt(C_5H_7N_3)_2Cl_2$ | Grey | 165 | - | 40.28 (40.26) | 17.35 (17.32) | 14.64 (14.62) | 484.22 (477.20) |
| $Pt(C_5H_5N_3)_2Cl_2Sn_2(CH_3)_4$ | Light Green | 141 | 25.08 (25.06) | 30.52 (30.49) | 10.80 (10.77) | 4.55 (4.52) | 777.68 (757.79) |
| $Pt(C_5H_5N_3)_2Cl_2Sn_2(C_6H_5)_4$ | Light Green | 139 | 19.01 (18.98) | 23.13 (23.10) | 8.19 (8.17) | 3.45 (3.42) | 1025.98 (1002.89) |
| $Pt(C_5H_5N_3)_2Cl_2Si_2(CH_3)_4$ | Light Green | 120 | 32.70 (32.68) | 9.41 (9.39) | 14.08 (14.05) | 11.88 (11.86) | 596.46 (579.41) |
| $Pt(C_5H_5N_3)_2Cl_2Si_2(C_6H_5)_4$ | Grey | 90 | 23.09 (23.07) | 6.65 (6.63) | 9.94 (9.91) | 8.39 (8.37) | 844.76 (829.43) |
| $Pt(C_5H_5N_3)_2Cl_2Ti_2(C_5H_5)_4$ | Brown | 136 | 23.32 (23.30) | 11.45 (11.42) | 10.04 (10.01) | 8.47 (8.44) | 836.32 (814.48) |
| $Pt(C_5H_5N_3)_2Cl_2Zr_2(C_5H_5)_4$ | Dark Green | 146 (d) | 21.13 (21.11) | 19.76 (19.73) | 9.10 (9.07) | 7.68 (7.66) | 923.00 (904.04) |

**Analytical Methods and Physical Measurements**

Conductivity measurements were made with a systronics model 305 conductivity bridge. The molecular weights were determined by the Rast camphor method. Infra red spectra of precursors and complexes were recorded in the range 4000-200 $cm^{-1}$ with the help of Nicolet-Megna FT-IR 550 spectrophotometer in KBr pellets. Electronic spectra were recorded on a Varian Cary/2390 spectrophotometer. $^1H$ NMR and metal NMR spectra were recorded in DMSO–$d_6$ versus TMS as standard on a JEOL FX–90Q spectrometer. Magnetic measurements of powdered samples at the room temperature were recorded on a vibrating sample magnetometer Model 155 at the RSIC, IIT, Madras. Platinum, titanum, zirconium, tin and silicon were estimated gravimetrically as their oxides. Nitrogen was estimated by Kjeldahl's method, and chlorine by Volhard's method.

**RESULTS AND DISCUSSION**

The elemental analysis and spectral data are consistent with the formulations of the compounds as $M(C_5H_7N_3)_2Cl_2$ and $[M(C_5H_5N_3)_2M'_2(R)_4]$. The reactions of $MCl_2$ with $C_5H_7N_3$ have been carried out in 1:2 molar ratios in methanol to yield $M(C_5H_7N_3)_2Cl_2$. This complex reacts with group four or fourteen metal dichloride to yield heterobimetallic chelates $[M(C_5H_5N_3)_2M'_2(R)_4]$.

The reactions proceed easily at room temperature. The coloured solid products so obtained are soluble in DMF and DMSO. The complexes are monomeric in camphor as indicated by the molecular weight determinations. Magnetic measurements showed them to be diamagnetic. However, the complexes are 1:2 electrolytes in DMSO as indicated by their molar conductance values (200-225 $ohm^{-1}$ $cm^2$ $mol^{-1}$).

***SPECTRAL STUDIES***

**IR Spectra**

The IR spectra of bimetallic complexes compare well the spectra of $M(C_5H_7N_3)_2Cl_2$. The primary stretch is located at higher frequency than that of the corresponding secondary amine[26]. The spectra of $M(C_5H_7N_3)_2Cl_2$ exhibit a broad and strong band in the range 3280 – 3140 $cm^{-1}$ assigned to υ(N-H). This band is found at lower frequency (3098 $cm^{-1}$)[27] in the complex. This may be taken as an evidence for the coordination of secondary nitrogen to metal. Aromatic ring stretching (c---c)[28] are present at 1646, 1520 and 1468 $cm^{-1}$. The presence of aromatic C-H and C-N bonds in the complex have been confirmed by the appearance of two bands at 3055 and 846 $cm^{-1}$, respectively.

The coordination of nitrogen to the metal is further supported by the appearance of new bands of medium to weak intensity in the regions 581 $cm^{-1}$ and 410 $cm^{-1}$ attributable to υ(Si ←N)[29] and υ(Sn ←N) vibrations, respectively. Apart from this, the band at 447 $cm^{-1}$ and 442 $cm^{-1}$ may be assigned to υ($Ti-C_5H_5$)[30] and υ ($Zr-C_5H_5$)[31] vibrations. The bands at *ca* 520-550 $cm^{-1}$ are due to υ(Ti-N)[32] and υ(Zr-N)[33] bonds.

**$^1H$ NMR Spectra**

The $^1H$ NMR spectra of the precursors and their complexes have been recorded in DMSO–$d_6$ using TMS as an internal standard. The disappearance of the amine proton signal and the appearance of a secondary amine proton signal in case of bimetallic complexes indicated the deprotonation of the $NH_2$ group after complexation. Chemical shift values of all the compounds are listed in Table II.

**Table II: $^1H$NMR Spectral Data (δ, ppm) of Precursors and Their Corresponding Complexes**

| Compound | $-NH_2$ | -NH | -R | -Ph |
|---|---|---|---|---|
| $Pd(C_5H_7N_3)_2Cl_2$ | 4.58 | - | - | 7.26-7.38 |
| $Pd(C_5H_5N_3)_2Cl_2Sn_2(CH_3)_4$ | - | 8.02 | 1.53 | 7.31-7.56 |
| $Pd(C_5H_5N_3)_2Cl_2Sn_2(C_6H_5)_4$ | - | 8.08 | 7.22-7.34 | 7.22-7.34 |
| $Pd(C_5H_5N_3)_2Cl_2Si_2(CH_3)_4$ | - | 8.13 | 1.67 | 7.04-7.51 |
| $Pd(C_5H_5N_3)_2Cl_2Si_2(C_6H_5)_4$ | - | 7.89 | 7.12-7.63 | 7.12-7.63 |
| $Pd(C_5H_5N_3)_2Cl_2Ti_2(C_5H_5)_4$ | - | 7.84 | 6.19 | 7.09-7.13 |
| $Pd(C_5H_5N_3)_2Cl_2Zr_2(C_5H_5)_4$ | - | 8.00 | 6.33 | 7.03-7.17 |
| $Pt(C_5H_7N_3)_2Cl_2$ | 4.56 | - | - | 7.09-7.23 |
| $Pt(C_5H_5N_3)_2Cl_2Sn_2(CH_3)_4$ | - | 7.98 | 1.28 | 7.01-7.21 |
| $Pt(C_5H_5N_3)_2Cl_2Sn_2(C_6H_5)_4$ | - | 8.04 | 7.09-7.37 | 7.09-7.37 |
| $Pt(C_5H_5N_3)_2Cl_2Si_2(CH_3)_4$ | - | - | 1.37 | 7.04-7.19 |
| $Pt(C_5H_5N_3)_2Cl_2Si_2(C_6H_5)_4$ | - | 8.02 | 7.04-7.35 | 7.04-7.35 |
| $Pt(C_5H_5N_3)_2Cl_2Ti_2(C_5H_5)_4$ | - | 8.00 | 6.51 | 7.06-7.15 |
| $Pt(C_5H_5N_3)_2Cl_2Zr_2(C_5H_5)_4$ | - | 7.93 | 6.42 | 7.00-7.29 |

**$^{119}Sn$, $^{29}Si$ and $^{195}Pt$ NMR Spectra**

The four coordination number of tin in these complexes were further get support by the appearance of sharp signals at δ–31.23ppm in $^{119}Sn$ NMR spectra[34].

In the cases of the silicon complexes, signals appeared at δ-50.99ppm are assigned for tetra coordinated state[35] around the silicon atom.

$^{195}Pt$ spectra show signals at δ-2721ppm, indicative to tetra coordinated state[36] of complexes.

**$^{13}C$ NMR Spectra**

In the $^{13}C$ NMR spectra of the precursors and their complexes, considerable shifts in the position of carbon atoms adjacent to atoms involved in complex formation clearly indicate the bonding of metal to the imino nitrogen atoms. Spectral data are given in Table III.

**Table III: $^{13}C$ NMR Spectral Data (δ,ppm) of Precursors and Their Corresponding Complexes.**

| Compound | C-M' | C-N | C3 | C4 | R |
|---|---|---|---|---|---|
| $Pd(C_5H_7N_3)_2Cl_2$ | - | 146.77 | 142.17 | 139.73 | - |
| $Pd(C_5H_5N_3)_2Cl_2Sn_2(CH_3)_4$ | 17.6 | 155.31 | 150.89 | 146.63 | - |
| $Pd(C_5H_5N_3)_2Cl_2Sn_2(C_6H_5)_4$ | 129.18 | 160.21 | 157381 | 153.62 | 127.15($C_2''$,$C_6''$), 125.93($C_3''$,$C_5''$), 124.58($C_4''$) |
| $Pd(C_5H_5N_3)_2Cl_2Si_2(CH_3)_4$ | 14.51 | 138.51 | 131.73 | 127.61 | - |
| $Pd(C_5H_5N_3)_2Cl_2Si_2(C_6H_5)_4$ | 134.62 | 152.30 | 150.83 | 141.98 | 132.60 ($C_2''$,$C_6''$),130.21 ($C_3''$,$C_5''$) 127.19 ($C_4''$) |
| $Pd(C_5H_5N_3)_2Cl_2Ti_2(C_5H_5)_4$ | 113.01 | 149.48 | 147.92 | 144.49 | 111.13 ($C_2'$, $C_5'$),101.98 ($C_3'$,$C_4'$) |
| $Pd(C_5H_5N_3)_2Cl_2Zr_2(C_5H_5)_4$ | 108.96 | 143.81 | 140.98 | 138.82 | 107.41 ($C_2'$,$C_5'$) 106.01 ($C_3'$,$C_4'$) |
| $Pt(C_5H_7N_3)_2Cl_2$ | - | 148.23 | 142.39 | 136.72 | - |
| $Pt(C_5H_5N_3)_2Cl_2Sn_2(CH_3)_4$ | 17.9 | 158.24 | 152.38 | 150.21 | - |
| $Pt(C_5H_5N_3)_2Cl_2Sn_2(C_6H_5)_4$ | 132.92 | 163.11 | 160.02 | 157.77 | 130.83($C_2''$,$C_6''$),128.81 ($C_3''$ $C_6''$) 127.91 ($C_4''$) |
| $Pt(C_5H_5N_3)_2Cl_2Si_2(CH_3)_4$ | 14.54 | 135.21 | 132.61 | 129.43 | - |
| $Pt(C_5H_5N_3)_2Cl_2Si_2(C_6H_5)_4$ | 136.83 | 153.39 | 151.30 | 146.83 | 134.98 ($C_2''$,$C_6''$),131.76($C_3''$,$C_5''$), 130.84 ($C_4''$) |
| $Pt(C_5H_5N_3)_2Cl_2Ti_2(C_5H_5)_4$ | 112.81 | 148.41 | 142.53 | 138.91 | 111.16 ($C_2'$,$C_5'$), 108.81 ($C_3'$,$C_4'$) |
| $Pt(C_5H_5N_3)_2Cl_2Zr_2(C_5H_5)_4$ | 109.74 | 144.12 | 140.09 | 138.82 | 108.81 ($C_2'$,$C_5'$), 106.93 ($C_3'$,$C_4'$) |

**Electronic Spectra**

The formation of heterobimetallic complexes is further supported by the electronic spectra. Palladium (II) and platinum(II) complexes display d-d spin-allowed transitions due to three lower lying 'd' levels to the empty $d_{x^2-y^2}$ orbitals. Transitions are located from ground state $^1A_1g$ to the exited states $^1A_2$, $^1B_1g$ and $^1Eg$ in order of increasing energy. Three d-d bands are assigned in the ranges 535 – 560nm, 455–475nm and 440 – 451nm in the palladium complexes and 519– 530nm, 440 – 465nm and 339– 376nm in the case of platinum complexes. These bands are attributed to $^1A_1g \rightarrow {}^1A_2g$, $^1A_1g \rightarrow {}^1B_1g$ and $^1A_1g \rightarrow {}^1Eg$ transitions, respectively. These spectral values support the square planar geometry (Fig.1) around Pd(II) and Pt(II) and are in agreement with those reported earlier for square planar complexes[37].

**BIOLOGICAL STUDIES**

Such complexes have served as models for a number of biochemical process. Therefore, all the complexes of palladium and platinum along with the precursors have been tested on various fungi and bacteria.

**Table IV: Fungicidal Screening Data of Precursors and Their Heterobimetallic Complexes (Percent growth inhibition after 4 days at 25±2°C, Conc. in ppm.)**

| Compound | *Fusarium oxysporum* | | | *Aspergillus niger* | | | *Helminthosporim gramineum* | | |
|---|---|---|---|---|---|---|---|---|---|
| | 100 | 150 | 200 | 100 | 150 | 200 | 100 | 150 | 200 |
| $Pd(C_5H_7N_3)_2Cl_2$ | 30 | 33 | 37 | 31 | 33 | 39 | 33 | 40 | 42 |
| $Pd(C_5H_5N_3)_2Cl_2Sn_2(CH_3)_4$ | 41 | 47 | 50 | 43 | 48 | 53 | 46 | 53 | 56 |
| $Pd(C_5H_5N_3)_2Cl_2Sn_2(C_6H_5)_4$ | 63 | 70 | 73 | 65 | 70 | 76 | 70 | 78 | 85 |
| $Pd(C_5H_5N_3)_2Cl_2Si_2(CH_3)_4$ | 38 | 39 | 42 | 39 | 43 | 47 | 44 | 49 | 53 |
| $Pd(C_5H_5N_3)_2Cl_2Si_2(C_6H_5)_4$ | 58 | 62 | 65 | 59 | 63 | 66 | 63 | 73 | 80 |
| $Pd(C_5H_5N_3)_2Cl_2Ti_2(C_5H_5)_4$ | 34 | 37 | 40 | 40 | 41 | 43 | 35 | 37 | 49 |
| $Pd(C_5H_5N_3)_2Cl_2Zr_2(C_5H_5)_4$ | 33 | 36 | 43 | 35 | 39 | 41 | 37 | 38 | 51 |
| $Pt(C_5H_7N_3)_2Cl_2$ | 31 | 32 | 35 | 32 | 33 | 35 | 35 | 39 | 43 |
| $Pt(C_5H_5N_3)_2Cl_2Sn_2(CH_3)_4$ | 43 | 46 | 53 | 46 | 49 | 56 | 48 | 52 | 57 |
| $Pt(C_5H_5N_3)_2Cl_2Sn_2(C_6H_5)_4$ | 66 | 73 | 77 | 68 | 70 | 78 | 72 | 79 | 83 |
| $Pt(C_5H_5N_3)_2Cl_2Si_2(CH_3)_4$ | 38 | 42 | 43 | 41 | 42 | 49 | 45 | 47 | 54 |
| $Pt(C_5H_5N_3)_2Cl_2Si_2(C_6H_5)_4$ | 61 | 70 | 72 | 62 | 69 | 72 | 69 | 73 | 78 |
| $Pt(C_5H_5N_3)_2Cl_2Ti_2(C_5H_5)_4$ | 35 | 36 | 40 | 38 | 39 | 40 | 39 | 43 | 50 |
| $Pt(C_5H_5N_3)_2Cl_2Zr_2(C_5H_5)_4$ | 33 | 37 | 41 | 37 | 39 | 43 | 38 | 44 | 47 |
| Standard (Bavistin) | 88 | 100 | 100 | 90 | 100 | 100 | 87 | 100 | 100 |

**Antifungal Activity**

The antifungal activity has been evaluated against several fungi by the radial growth method[38]. The compounds were directly mixed with the medium in 100, 150 and 200 ppm concentrations. Controls were also run and three replicates were used in each case. The linear growth of the fungus was obtained by measuring the diameter of the fungal colony after four days. The amount of growth inhibition in each of the replicates was calculated by equation (C-T) 100 $C^{-1}$, where C is the diameter of the colony on the control plate and T is the diameter of the fungal colony on the test plate.

**Antibacterial Activity**

The antibacterial activity was determined by the inhibition zone technique. All the compounds were dissolved in methanol, paper discs of Whatman No. 1 paper with a diameter of 5mm were soaked in these solutions. These discs were placed on the appropriate medium previously seeded with organisms in Petri discs and stored in an incubator at 30±1$^0$C. The inhibition zone thus formed around each disc was measured in mm after 24 hours. Data of these activities are summarised in Tables IV and V.

**Table V: Bactericidal Screening Data of Precursors and Their Heterobimetallic Complexes (Dimeter of inhibition zone (mm) after 24 hours at 30±1$^0$C)**

| Compound | *Escherichia coli* | | *Klebsiella aerogenous* | | *Pseudomonas cepacicola* | |
|---|---|---|---|---|---|---|
| | 500 | 1000 | 500 | 1000 | 500 | 1000 |
| $Pd(C_5H_7N_3)_2Cl_2$ | 3 | 4 | 2 | 4 | 2 | 3 |
| $Pd(C_5H_5N_3)_2Cl_2Sn_2(CH_3)_4$ | 6 | 10 | 7 | 9 | 7 | 8 |
| $Pd(C_5H_5N_3)_2Cl_2Sn_2(C_6H_5)_4$ | 9 | 11 | 10 | 11 | 11 | 13 |
| $Pd(C_5H_5N_3)_2Cl_2Si_2(CH_3)_4$ | 4 | 5 | 5 | 7 | 4 | 6 |
| $Pd(C_5H_5N_3)_2Cl_2Si_2(C_6H_5)_4$ | 7 | 8 | 9 | 10 | 9 | 11 |
| $Pd(C_5H_5N_3)_2Cl_2Ti_2(C_5H_5)_4$ | 4 | 5 | 6 | 7 | 5 | 6 |
| $Pd(C_5H_5N_3)_2Cl_2Zr_2(C_5H_5)_4$ | 4 | 6 | 6 | 6 | 6 | 7 |
| $Pt(C_5H_7N_3)_2Cl_2$ | 2 | 3 | 2 | 4 | 3 | 4 |
| $Pt(C_5H_5N_3)_2Cl_2Sn_2(CH_3)_4$ | 7 | 8 | 6 | 7 | 5 | 8 |
| $Pt(C_5H_5N_3)_2Cl_2Sn_2(C_6H_5)_4$ | 11 | 12 | 8 | 10 | 9 | 11 |
| $Pt(C_5H_5N_3)_2Cl_2Si_2(CH_3)_4$ | 5 | 6 | 4 | 5 | 4 | 6 |
| $Pt(C_5H_5N_3)_2Cl_2Si_2(C_6H_5)_4$ | 9 | 10 | 7 | 9 | 7 | 10 |
| $Pt(C_5H_5N_3)_2Cl_2Ti_2(C_5H_5)_4$ | 4 | 4 | 3 | 5 | 4 | 5 |
| $Pt(C_5H_5N_3)_2Cl_2Zr_2(C_5H_5)_4$ | 4 | 5 | 5 | 6 | 4 | 5 |
| Standard (Streptomycin) | 17 | 18 | 13 | 14 | 15 | 16 |

The results reveal that the activity increases on complexation. The newly synthesized complexes have indeed been found to be more active in inhibiting the growth of fungi and bacteria than the precursors themselves. It may be postulated that these complexes might act as uncoupling agents of oxidation posphorylation. The first uncoupling agent to be described, by Loomis and Lipmann[39] was 2,4-dinitrophenol. Today many different uncoupling agents are known. Most are lipid soluble substances containing an acidic group and usually an aromatic ring. These agents allow electron transport to continue but prevent the phosphorylation of ADP to ATP. They uncouple the energy-yielding from the energy conserving reactions. Uncoupling agents function by breaking down a high-energy intermediate or state generated by electron transport. They can promote the passage of $H^+$ ions through the cell membrane, which is normally impermeable to them[40]. However, these agents are less effective for bacteria. The greater toxicity of metal complexes than the precursors can also be explained on the basis of the chelation theory[41,42]. Chelation reduces the polarity of metal ion mainly because of partial sharing of its positive charge with the donor groups and possible π-electron–delocalisation over the whole chelation ring. This increases the lipophilic character of the metal complex, which subsequently favours its permeation through the lipid layers of the organism cell membrane, and the normal cell process being impaired.

**Antifertility Activity**

Male rats obtained from ICMR, New Delhi were used. Animals were housed in steel cages and maintained under standard conditions (12 h light/ 12 h dark cycle; 25 ±3$^0$C, 35 – 60% humidity), water and food were given *ad libitum*. Proven fertile male rats were taken and divided into nine groups of six each. The group A served as vehicle (olive oil) treated control. For groups B and F, starting material ($PdCl_2$, 50mg/kg.b.wt.) suspended in olive oil was given for a period of 60 days. The animals of groups C, D and E received same dose of its $Pd(C_5H_7N_3)_2Cl_2$, $[Pd(C_5H_5N_3)_2Sn_2(CH_3)_4]Cl_2$ and $[Pd(C_5H_5N_3)_2Sn_2(C_6H_5)_4]Cl_2$ respectively for the same period. The animals of group F received starting material that is $PtCl_2$ 50mg/kg.b.wt. suspended in olive oil. The animals of groups G,H and I received $Pt(C_5H_7N_3)_2Cl_2$, $[Pt(C_5H_5N_3)_2Sn_2(CH_3)_4]Cl_2$ and $[Pt(C_5H_5N_3)_2Sn_2(C_6H_5)_4]Cl_2$ respectively for the same period and dose. On the day sixty first, these animals were autopsied and testes, epididymis, seminal vesicle and ventral prostate were removed, fat and connective tissue cleared off and kept at $-20^0C$ until assayed for total protein, sialic acid, cholestrol, fructose and glycogen by standard laboratory techniques.

**Table VI: Alteration in the Body Weight and Weight of Reproductive Organs after Treatment with Various Compounds**

| Group | Treatment | Body Weight (g) | | Testes mg/100 gm b.wt. | Epididymis mg/100 gm b.wt. | Seminal Vesicle mg/100 gm b.wt. | Ventral Prostrate mg/100 gm b.wt. |
|---|---|---|---|---|---|---|---|
| | | Initial | Final | | | | |
| A | Control | 180 ±15 | 201±18$^c$ | 1210±90 | 480±30 | 360±20 | 255±15$^a$ |
| B | $PdCl_2$ | 175±12 | 200±10$^c$ | 900±80$^a$ | 305±22$^b$ | 280±12$^b$ | 210±9b$^a$ |
| C | $Pd(C_5H_7N_3)_2Cl_2$ | 178±20 | 185±10$^c$ | 805±50$^a$ | 270±15$^a$ | 240±12$^a$ | 180±12$^a$ |
| D | $Pd(C_5H_5N_3)_2Cl_2Sn_2(CH_3)_4$ | 185±11 | 198±12$^c$ | 705±50$^a$ | 210±18$^a$ | 215±13$^b$ | 140±15$^b$ |
| E | $Pd(C_5H_5N_3)_2Cl_2Sn_2(C_6H_5)_4$ | 190±15 | 215±10$^c$ | 700±70$^a$ | 200 ±19$^b$ | 175±18$^a$ | 130±10$^b$ |
| F | $PtCl_2$ | 192±18 | 220±12$^c$ | 910±70$^b$ | 320±20$^b$ | 275±14$^b$ | 200±18$^a$ |
| G | $Pt(C_5H_7N_3)_2Cl_2$ | 183±20 | 218±15$^c$ | 720±80$^a$ | 260±14$^a$ | 230±19$^a$ | 170±20$^a$ |
| H | $Pt(C_5H_5N_3)_2Cl_2Sn_2(CH_3)_4$ | 179±15 | 199±18$^c$ | 650±70$^a$ | 208±15$^a$ | 200±22$^a$ | 143±25$^b$ |
| I | $Pt(C_5H_5N_3)_2Cl_2Sn_2(C_6H_5)_4$ | 181±12 | 205±10$^c$ | 630±50$^a$ | 195±18$^b$ | 170±25$^b$ | 120±22$^b$ |

a = $p\leq 0.05$ b = $p\leq 0.001$ c = p = NS

Groups B and F compared with group A
Groups D and E compared with group C
Groups H and I compared with group G
Group C compared with group B
Group G compared with group F

**Table VII: Altered Sperm Dynamics and Fertility Test after Treatment with Precursors and Their Complexes**

| Group | Treatment | Sperm motility (%) Cauda epididymis | Sperm Density (Million/ml) | | Fertility Test (%) |
|---|---|---|---|---|---|
| | | | Testes | Cauda Epididymis | |
| A | Control | 74±3.0 | 3.5±0.21 | 55±4 | 100% (positive) |
| B | $PdCl_2$ | 60±3.5$^b$ | 2.0±0.5$^b$ | 39±2$^b$ | 80% (negative) |
| C | $Pd(C_5H_7N_3)_2Cl_2$ | 50±3.1$^a$ | 1.1±0.5$^a$ | 35±1.5$^b$ | 85% (negative) |
| D | $Pd(C_5H_5N_3)_2Cl_2Sn_2(CH_3)_4$ | 45±2.5$^a$ | 1.0±0.4$^a$ | 20±1.8$^b$ | 90% (negative) |
| E | $Pd(C_5H_5N_3)_2Cl_2Sn_2(C_6H_5)_4$ | 40±3.2$^a$ | 0.8±0.17$^a$ | 19±0.9$^b$ | 95% (negative) |
| F | $PtCl_2$ | 62±2.8$^{ba}$ | 2.7±0.6$^a$ | 50±5$^b$ | 75 % (negative) |
| G | $Pt(C_5H_7N_3)_2Cl_2$ | 55±2.4$^a$ | 1.2±0.4$^b$ | 40±2$^b$ | 82% (negative) |
| H | $Pt(C_5H_5N_3)_2Cl_2Sn_2(CH_3)_4$ | 49±2.2$^a$ | 0.9±0.2$^b$ | 18±3$^b$ | 88% (negative) |
| I | $Pt(C_5H_5N_3)_2Cl_2Sn_2(C_6H_5)_4$ | 41±3.1$^a$ | 0.7±0.10 | 15±2$^b$ | 98% (negative) |

See footnote of Table VI

**Fertility Test**

The mating exposure tests of all the animals were performed from day $55^{th}$ to $60^{th}$. They were cohabitated with proestrous females in the ratio 1:3. The vaginal plug and the presence of sperm in the vaginal smear were checked for positive mating. The mated females were separated to note the implantation sites on day $16^{th}$ of pregnancy through leprotomy.

**Body and Organ Weight**

No significant change was observed in the body weight after treatment with the compounds. A significant reduction in the weight of testes, epididymis, seminal vesicle and ventral prostate was observed after treatment with both the precursors and the compounds (Table VI).

**Sperm Dynamics**

Sperm motility in cauda epididymis and sperm density in testes and cauda epididymis were significantly reduced after treatment with both the precursors and their compounds (Table VII).

### BIOCHEMICAL PARAMETERS LEADING TO INFERTILITY

**Total Protein**

Treatment with both the precursors as well as their complexes resulted in a significant reduction in the total protein contents of testes, epididymis, seminal vesicle and ventral prostate (Table VIII).

**Table VIII: Effects of Precursors and Their Complexes on Total Protein Contents of Various Reproductive Organs of Male Rats**

| Group | Treatment | Total Protein (mg/gm) | | | |
|---|---|---|---|---|---|
| | | Testes | Epididymis | Seminal Vesicle | Ventral prostate |
| A | Control | 190±10 | 255±10 | 230±5 | 240±12 |
| B | $PdCl_2$ | 150±5 [a] | 190±20 [a] | 180±4 [b] | 185±7 [b] |
| C | $Pd(C_5H_7N_3)_2Cl_2$ | 130±6 [a] | 140±10 [a] | 130±5 [b] | 138±8 [b] |
| D | $Pd(C_5H_5N_3)_2Cl_2Sn_2(CH_3)_4$ | 105±10 [b] | 102±8 [b] | 120±7 [b] | 114±9 [b] |
| E | $Pd(C_5H_5N_3)_2Cl_2Sn_2(C_6H_5)_4$ | 100±12 [b] | 105±10 [b] | 105±8 [b] | 109±10 [b] |
| F | $PtCl_2$ | 155±4 [a] | 200±8 [a] | 185±9 [a] | 190±8 [b] |
| G | $Pt(C_5H_7N_3)_2Cl_2$ | 145±8 [a] | 150±3 [b] | 128±7 [b] | 150±6 [b] |
| H | $Pt(C_5H_5N_3)_2Cl_2Sn_2(CH_3)_4$ | 110±4 [b] | 104±2 [b] | 111±6 [b] | 128±6 [b] |
| I | $Pt(C_5H_5N_3)_2Cl_2Sn_2(C_6H_5)_4$ | 103±5 [b] | 105±5 [b] | 101±4 [b] | 11±10 [b] |

See footnote of Table VI

**Sialic Acid**

A significant reduction in sialic acid contents of testes, epididymis, seminal vesicle and ventral prostate was observed after the treatment in all experimental groups (Table IX).

**Table IX: Effects of Precursors and Their Complexes on Sialic Acid of Various Reproductive Organs of Male Rats**

| Group | Treatment | Sialic Acid (mg/gm) | | | |
|---|---|---|---|---|---|
| | | Testes | Epididymis | Seminal Vesicle | Ventral prostate |
| A | Control | 8.5±0.9 | 7.3±0.8 | 7.9±0.6 | 8.1±0.8 |
| B | $PdCl_2$ | 6.1±0.7 [a] | 5.3±0.9 [a] | 6.1±0.2 [a] | 6.3±0.2 [b] |
| C | $Pd(C_5H_7N_3)_2Cl_2$ | 4.6±0.4 [b] | 4.4±0.5 [b] | 4.1±0.1 [b] | 5.0±0.1 [a] |
| D | $Pd(C_5H_5N_3)_2Cl_2Sn_2(CH_3)_4$ | 4.3±0.2 [b] | 3.8±0.6 [b] | 3.4±0.3 [b] | 4.1±0.2 [b] |
| E | $Pd(C_5H_5N_3)_2Cl_2Sn_2(C_6H_5)_4$ | 3.9±0.2 [b] | 3.7±0.4 [b] | 3.1±0.2 [b] | 4.0±0.1 [b] |
| F | $PtCl_2$ | 5.9±0.5 [b] | 5.7±0.4 [b] | 5.8±0.3 [b] | 6.2±0.1 [b] |
| G | $Pt(C_5H_7N_3)_2Cl_2$ | 3.8±0.1 [b] | 4.0±0.3 [b] | 3.8±0.5 [b] | 4.9±0.1 [b] |
| H | $Pt(C_5H_5N_3)_2Cl_2Sn_2(CH_3)_4$ | 3.5±0.2 [b] | 3.6±0.2 [b] | 3.2±0.1 [b] | 4.2±0.3 [b] |
| I | $Pt(C_5H_5N_3)_2Cl_2Sn_2(C_6H_5)_4$ | 3.0±0.3 [b] | 3.1±6.1 [b] | 3.0±0.2 [b] | 3.4±0.5 [b] |

See footnote of Table VI

**Cholesterol**

Cholesterol contents of testes were decreased significantly in all experimental groups (Table X).

**Fructose**

A significant decrease in the seminal vesicular fructose was noticed in all experimental groups (Table IX).

**Table X: Effects of Precursors and Their Complexes on Tissue Cholesterol Glycogen and Fructose**

| Group | Treatment | Fructose (mg/gm) Seminal Vesicle | Cholesterol (mg/gm) Testes | Glycogen (mg/gm) Testes |
|---|---|---|---|---|
| A | Control | 450±30 | 8.2±0.6 | 5.0±0.3 |
| B | $PdCl_2$ | 360±10 [b] | 6.2±0.5 [b] | 3.7±0.2 [b] |
| C | $Pd(C_5H_7N_3)_2Cl_2$ | 290 ±15 [b] | 5.1±0.1 [b] | 2.9±0.2 [b] |
| D | $Pd(C_5H_5N_3)_2Cl_2Sn_2(CH_3)_4$ | 280±10 [b] | 4.9±0.2 [b] | 2.5±0.3 [b] |
| E | $Pd(C_5H_5N_3)_2Cl_2Sn_2(C_6H_5)_4$ | 260±12 [b] | 4.4±0.3 [b] | 2.0±0.1 [b] |
| F | $PtCl_2$ | 375±15 [b] | 6.7±0.2 [b] | 3.2±0.1 [b] |
| G | $Pt(C_5H_7N_3)_2Cl_2$ | 300±5 [b] | 3.8±0.5 [b] | 2.1±0.2 [b] |
| H | $Pt(C_5H_5N_3)_2Cl_2Sn_2(CH_3)_4$ | 275±12 [b] | 3.5±0.3 [b] | 2.2±0.2 [b] |
| I | $Pt(C_5H_5N_3)_2Cl_2Sn_2(C_6H_5)_4$ | 220±20 [b] | 3.1±0.2 [b] | 2.4±0.1 [b] |

Cf. Table VI

**Glycogen**

Testicular glycogen was depleted significantly in all experimental groups (Table IX).

Present study showed that oral administration of $PdCl_2$, $PtCl_2$, precursors and their complexes resulted in the reduction of weights of testes, epididymis, seminal vesicle and ventral prostate. The weight, size and secretory activities of sex accessories are closely regulated by androgen levels[43]. Reduction in sperm density and motility in cauda epididymis is of importance with regards to fertilization[44]. Significant reduction in the sperm motility and sperm density was observed in treated animals. This may be due to inhibitory effect of these compounds on the enzyme oxidative phosphorylation[45]. In our study various androgen dependent parameters that is total protein, sialic acid, fructose, cholestrol and glycogen revealed a significant decrease indicating that administration of these compunds resulted in the fall of circulating androgen[46,47]. It is inferred that compounds of Pd and Pt are found to be more effective than the starting materials in inhibiting the fertility.

### ACKNOWLEDGEMENT

The authors are thankful to CSIR, New Delhi, India for financial assistance through grant number 01(1490)/EMR – II.

### REFERENCES

1. E. Kimura, M. Shinoya, A. Hoshino, T. Ireda and Y. Yamada, *J. Am. Chem. Soc.*, **114,** 10134 (1992).
2. J. G. Muller, X. Chem, A. C. Dadiz, S. E. Rokita and C. S. Burrows, *Pure Appl. Chem.,* **65,** 545 (1993).
3. K. J. Lee and J. Suh, *Bioorg. Chem,* **22,** 95 (1994).
4. T. M. Garrett, T. J. McMurry, M. W. Hosseini, Z.E. TReyeo, F.E. Hahn and K.N. Raymond, *J. Am. Chem. Soc.,* **113,** 2965 (1991).
5. G. De Santis, L. Fabbrizzi, M. Licchelli, C. Mangano, P. Pallavichini and A. Poggi, *Inorg. Chem.*, **32,** 834 (1993).
6. R. D. Hancock, G. Pattrick, P. W. Wade and G. D. Hosken, *Pure Appl. Chem.*, **65,** 473 (1993).
7. R. L. Webb, M. L. Mino, E. L. Blinn and A. A. Pinkerton, *Inorg. Chem.,* **32,** 1396 (1993).
8. S. G. Kang, M.S. Kim, D. Whang and K. Kim, *J. Chem. Soc. Dalton Trans,* 853 (1994).
9. E. S. Eggleston and S. C. Jackels, *Inorg. Chem.*, **19,** 1593 (1980).
10. G. B. D. Micheal, C. Yales Paul, P. M. Brain, N. Jane and S. M. Neleon, *Inorg. Chim. Acta,* **118,** 37 (1986).
11. C. Saxena and R. V. Singh, *Phosphorous, Sulfur and Silicon,* **97,** 17 (1994).
12. H. Naggy Kovacs, A.D. Delman and B. B. Simms, *J. Polymer Sci.*, **4,** 1081 (1966).
13. V. Bazant, V. Chvalovsky and J. Rathousky, *Organosilicon Compounds*, (Czech. Acad. Sci., Prague and Academic Press, New York) **2,** Parts 1 and 2 (1965).
14. P. J. Smith and D.Dodd, *J. Organomet. Chem.*, **32,** 195 (1975).
15. R. Barbieri, G. Alonzo, A. Silvestri, N. Burriocsci, N., Bertazzi, G. C. Stoeco and L. Pellerito, *Gazz. Chem., It.* **104,** 885 (1974).
16. R. Willem, H. Dalil, P. Broeckaert, M. Biesemans, L. Ghys, K. Nooter, D.de Vos, F. Ribot and M. Gielen, *Main Group Met. Chem.*, **20,** 535 (1997).
17. S.W. Ng., J.M. Hook and M. Gielen, *Appl. Organomet. Chem.*,**14,** 1 (2000).
18. A. J. Crowe, *Drugs of the Future*, **3,** 225 (1987).
19. S. Belwal, R. K. Saini and R. V. Singh, *Indian. J. Chem.*, **37A,** 245 (1998).
20. K. Dey, D. Bandyopadhyay, K. K. Nandi, S. N. Poddar, G. Mukhopadhyay and G. B. Kauffman, *Synth. React. Inorg. Met. – Org. Chem.,* **22,** 1111 (1992).
21. M. A. Aliand S. E. Livingstone, *Coord. Chem. Rev.,* **13,** 101 (1974).
22. I. Haiduc, *Coord. Chem. Rev.,* **99,** 253 (1990) and references therein.
23. M. J. Cleare, *Coord. Chem. Rev.,* **12,** 349 (1974).
24. B. Singh, R. N. Singh and R. C. Aggarwal, *Polyhedron,* **4,** 401 (1985).
25. S.Wang, Garzon, C.King, J. Wang, and J.P. Fackler Jr., *Inorg. Chim. Acta,* **62,** 57 (1982).
26. K. Nakamato, *Infrared and Raman Spectra of Inorganic and Coordination Compounds*, Willey, New York, **201,** (1978)
27. K. S. Siddiqui, F. M. A. M. Aqra and S. A. A. Zaidi, *Trans. Met. Chem.,***18,** 421 (1993).
28. R. M. Silverstein, G. S. Bassler, and T. C. Morrill, *Spectrometric Identification of Organic Compounds* $4^{th}$ Ed. (1981), p. 112
29. E. A. V. Ebsworth and M. J. Mays, *J. Chem. Soc.*,3750 (1964).
30. R.S.P. Coutts and P.C. Wailes, *J. Organomet. Chem.,* **84,** 47 (1975).
31. K. Chandra, R. K. Sharma, B.S. Garg and R.P. Singh, *Transition Met. Chem.*, **4,** 367 (1979).
32. N. S. Biradar and A. L. Locker, *J. Inorg. Nucl. Chem.*, **36,** 1915 (1974).
33. N. H. Khan, K. S. Siddiqi, R. I. Kureshy, S. Tabassum and S. S. A. Zaidi, *Indian J. Chem.*, **26A,** 763 (1987).
34. R. Colton and D. Dakternieks, *Inorg. Chim. Acta*, **148,** 32 (1988).
35. R. K. Sharma, Y. P. Singh and A. K. Rai, *Indian J. Chem.*, **38 A,** 604 (1999).
36. A. Irving, K. R. Koch and M. Motoetoe, *Inorg. Chim., Acta*, **206,** 195 (1993).

37. H. B. Gray and C. J. Ballhausen, *J. Am. Chem. Soc.,* **85**, 260 (1963).
38. D. Singh, R. B. Goyal and R. V. Singh, *Appl. Organomet. Chem.,* **5,** 45 (1991).
39. N. Fahmi, S. C. S. Jadon and R. V. Singh, *Phosphorus, Sulfur and Silicon*, **81**, 137 (1993).
40. A. L. Lehninger, "*Biochemistry*", Second Edition, 519 (1975).
41. R. S. Srivastava, *Inorg. Chim. Acta,* **56,** 165 (1981).
42. K. N. Thimmaich, W. D. Lloyd and G. T. Chandrappa, *Inorg. Chim. Acta*, **106**, 81 (1985).
43. M. Chaturvedi and V. P. Dixit, *J. Environmental Sci.*, **1,** 89 (1997).
44. J. M. Bedford, *Biol. Reprod.,* **28**, 108 (1983).
45. A. K. Purohit, *Bio. Sci.,* **3,** 179 (1991).
46. S. Belwal, S. C. Joshi and R. V. Singh, *Main Group Met. Chem.,* **20**, 313 (1997).
47. P. C. Mali, *Indian J. Environmental Sci.,* **3,** 185 (1999).

**Received: January 25, 2000 - Accepted: February 2, 2000 -**
**Received in revised camera-ready format: April 27, 2000**

